# Enhancing fraud detection in banking by integration of graph databases with machine learning

**DOI:** 10.1016/j.mex.2024.102683

**Published:** 2024-04-04

**Authors:** Ayushi Patil, Shreya Mahajan, Jinal Menpara, Shivali Wagle, Preksha Pareek, Ketan Kotecha

**Affiliations:** a Artificial Intelligence & Machine Learning Department, Symbiosis Institute of Technology, Symbiosis International (Deemed University), Pune, Maharashtra 412115, India; b Symbiosis Centre for Applied Artificial Intelligence, Symbiosis Institute of Technology, Symbiosis International Deemed University Maharashtra, Pune 412115, India

**Keywords:** Graph based fraud detection, Fintech, Bank transactions, Machine learning algorithms, Fraud detection, Online banking

## Abstract

The banking sector's shift from traditional physical locations to digital channels has offered customers unprecedented convenience and increased the risk of fraud for customers and institutions alike. In this study, we discuss the pressing need for robust fraud detection & prevention systems in the context of evolving technological environments. We introduce a graph-based machine learning model that is specifically designed to detect fraudulent activity in various types of banking operations, such as credit card transactions, debit card transactions, and online banking transactions. This model uses advanced methods for anomalies, behaviors, and patterns to analyze past transactions and user behavior almost immediately. We provide an in-depth methodology for evaluating fraud detection systems based on parameters such as Accuracy Recall rate and False positive rate ROC curves. The findings can be used by financial institutions to develop and enhance fraud detection strategies as they demonstrate the effectiveness and reliability of the proposed approach. This study emphasizes the critical role that innovative technologies play in safeguarding the financial sector from the ever-changing strategies of fraudsters while also enhancing banking security.

• This paper aims to implement the detection of fraudulent transactions using a state-of-the-art Graph Database approach.
• The relational graph of features in the dataset used is modelled using Neo4J as a graph database.
• Applying JSON features from the exported graph to various Machine Learning models, giving effective outcomes.

## Graphical abstract

**Figure:**
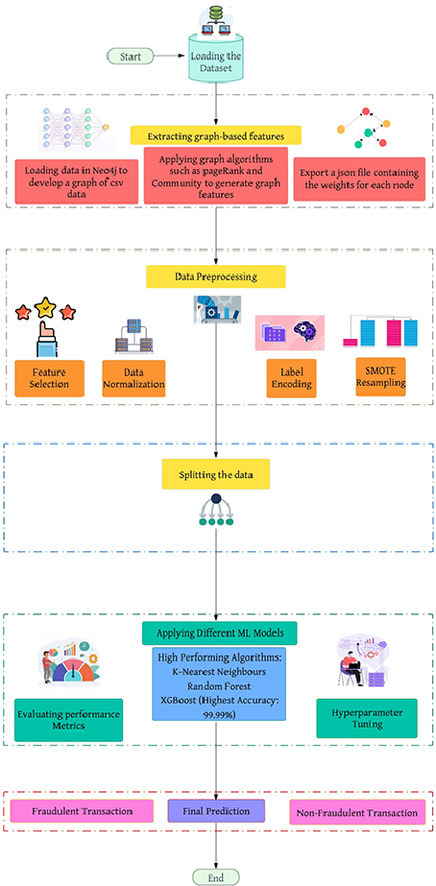

Specifications table

**Table:** 

Subject area	Engineering
More specific subject area	Finance with AI
Name of your method	Graph based fraud detection
Name and reference of original method	*PageRank Algorithm* *Prusti, D., Das, D. & Rath, S.K. Credit Card Fraud Detection Technique by Applying Graph Database Model. Arab J Sci Eng 46, 1–20 (2021).*https://doi.org/10.1007/s13369–021–05682–9
Resource availability	*Data:*https://www.kaggle.com/datasets/ealaxi/banksim1 Software: Google Colab

## Method details

**Fig. 1:**
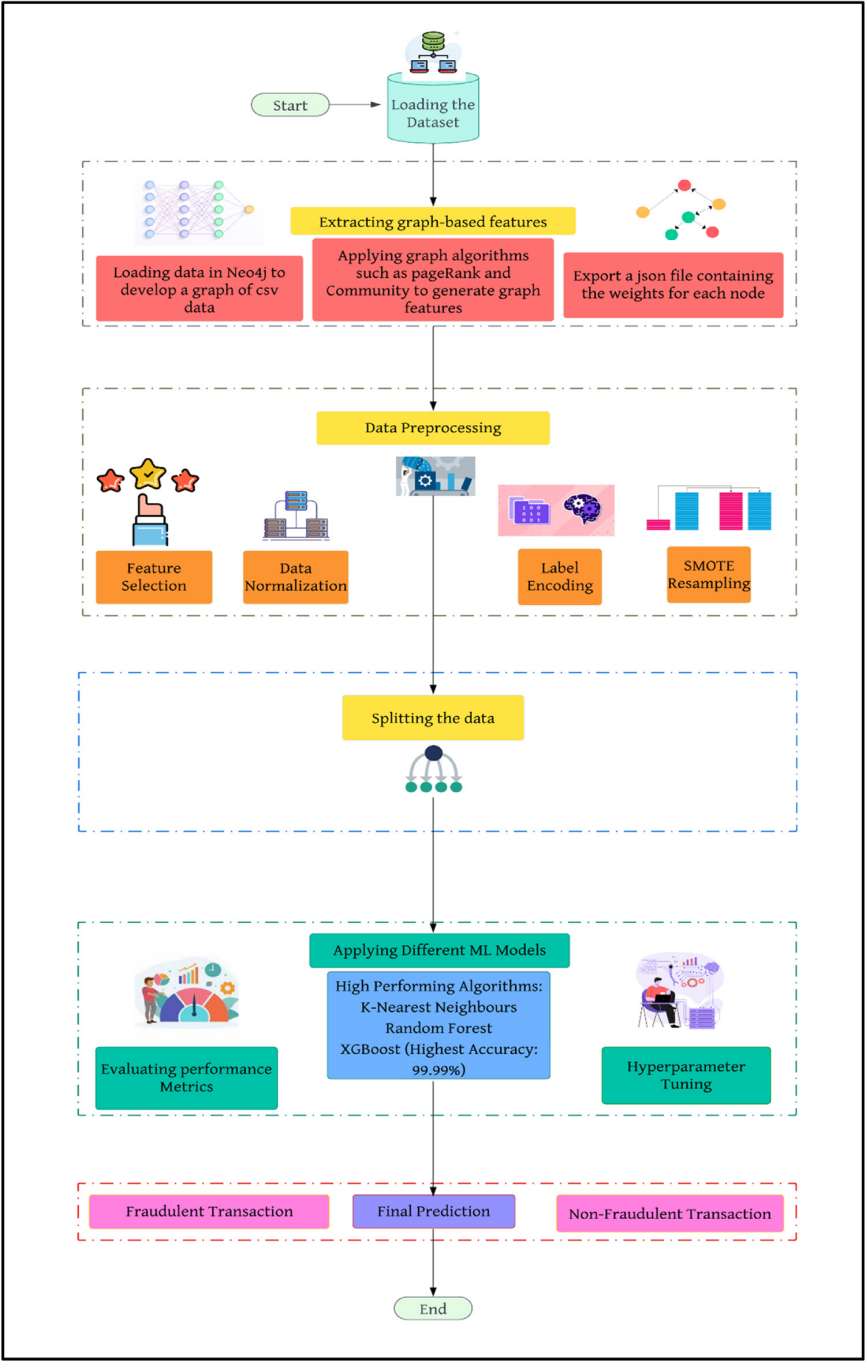
Proposed flow of methodology.

In the proposed methodology as described in Fig. 1, in brief, we have loaded our dataset, commonly known as ‘Banksim’, collected from Kaggle into a graph database. Then we have applied various graph functions through which we have extracted graph-based features to further preprocess the data. In the data preprocessing step, we have first selected the features that are relevant to our implementation and normalised them in a standard numeric range. Besides, we have also converted the categorical features into numerical data using data encoding tools such as One Hot encoding. Furthermore, we found that our data was imbalanced, hence we have used SMOTE as a resampling method to balance both categories in the data. After this step, we followed the standard ML pipeline of splitting the data and applying various ML models on our data.

The first step is to load the dataset into our system, setting the scene for the upcoming steps. The dataset contains a large number of features, each containing valuable information. To leverage the power of graph insights, we have used an advanced querying technique using a graph database named Neo4J.

Graphs are designed to operate on connected information. A graph data model's nodes, or vertices, and edges, or links, are its fundamental building blocks. An individual data point, such as a person, place, phone number, etc., is represented by a node. An edge is the connection between two nodes, such as a phone number. Nodes and the relationships that go with them are stored together in a graph model, so all we have to do for real-time queries is follow the arrows. We can generate predictions about your graph using graph embeddings and graph database machine learning training inside the analytics workspace, owing to the Neo4j graph algorithms that examine global structures to identify significant trends.

**Fig. 2:**
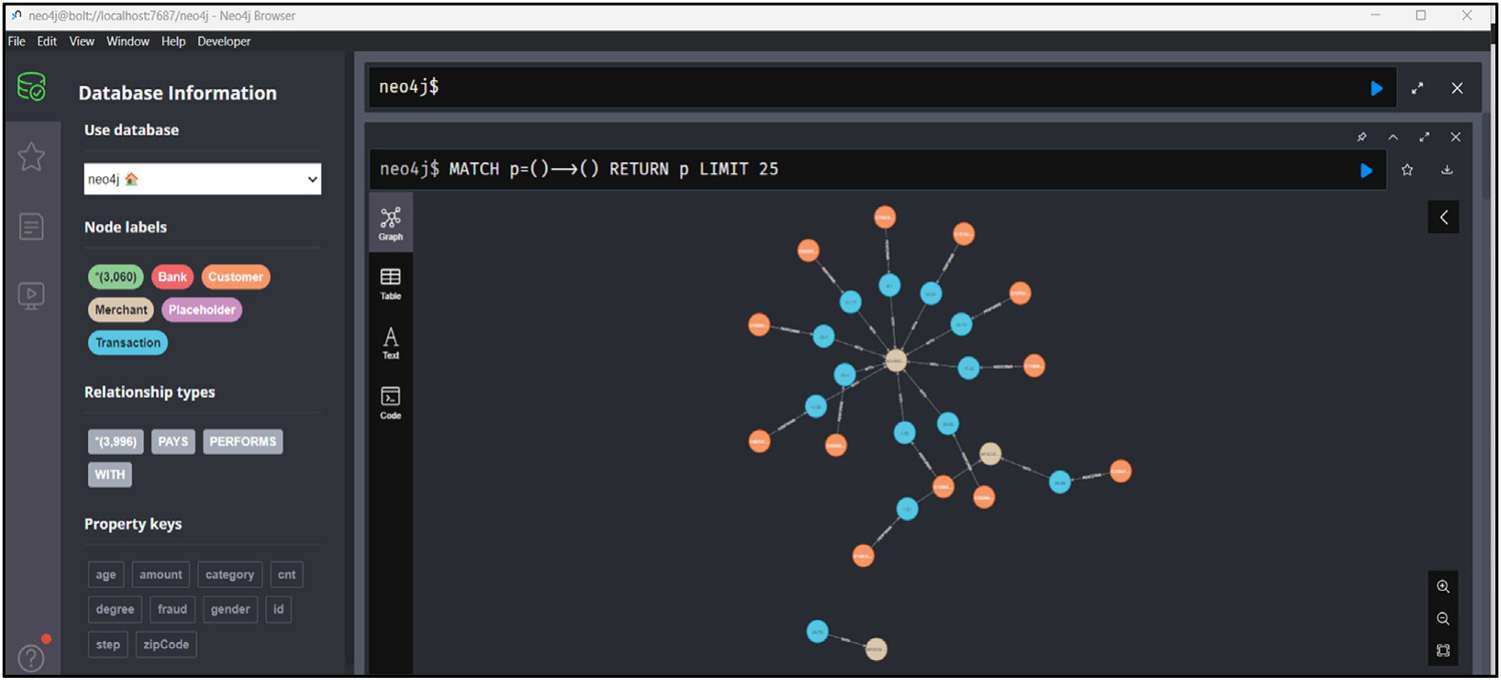
Neo4j Browser Overview.

**Fig. 3:**
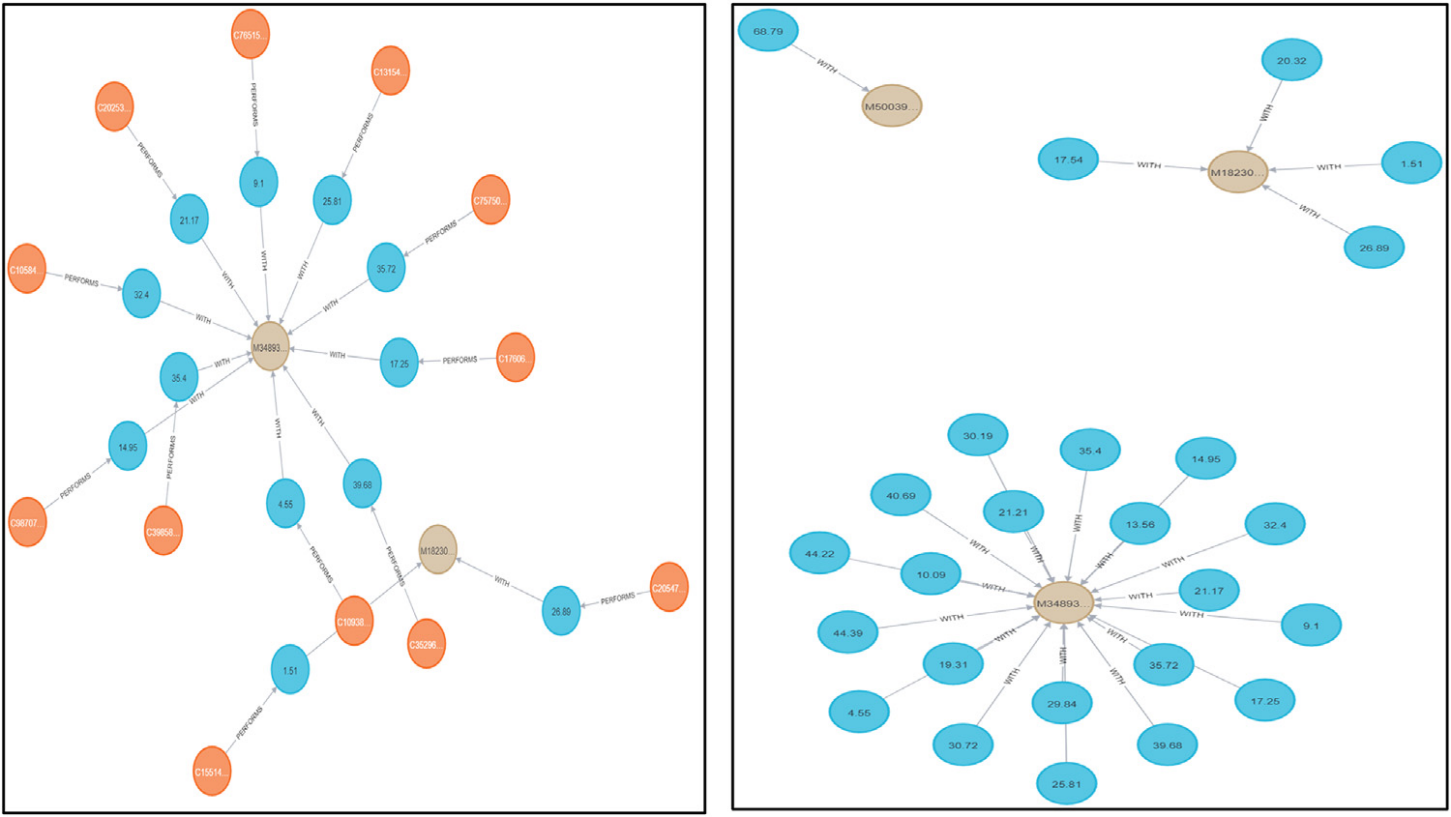
Neo4j graphs for relationships in the dataset (‘PERFORMS’ and ‘WITH’).

**Fig. 4:**
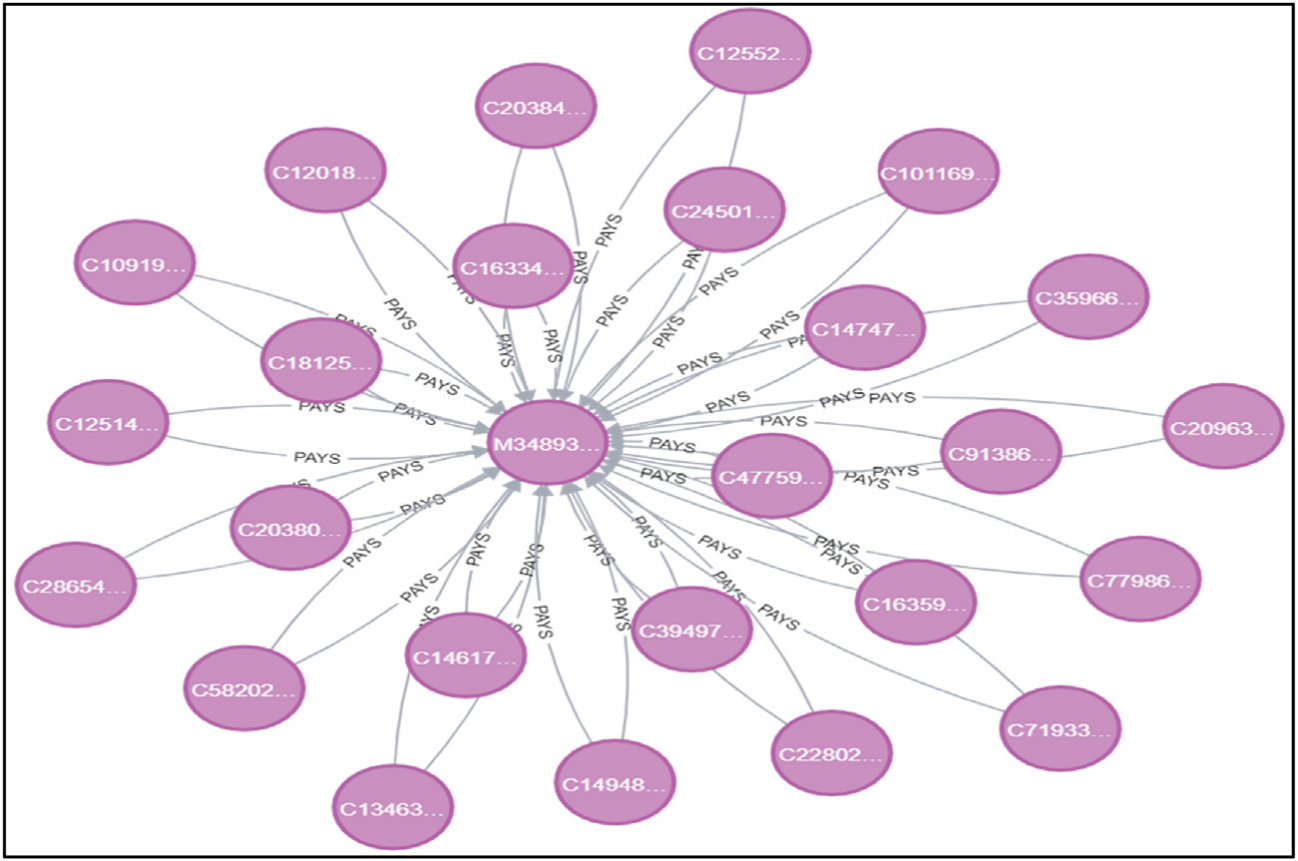
Graph for ‘PAYS’ relationship.

**Fig. 5:**
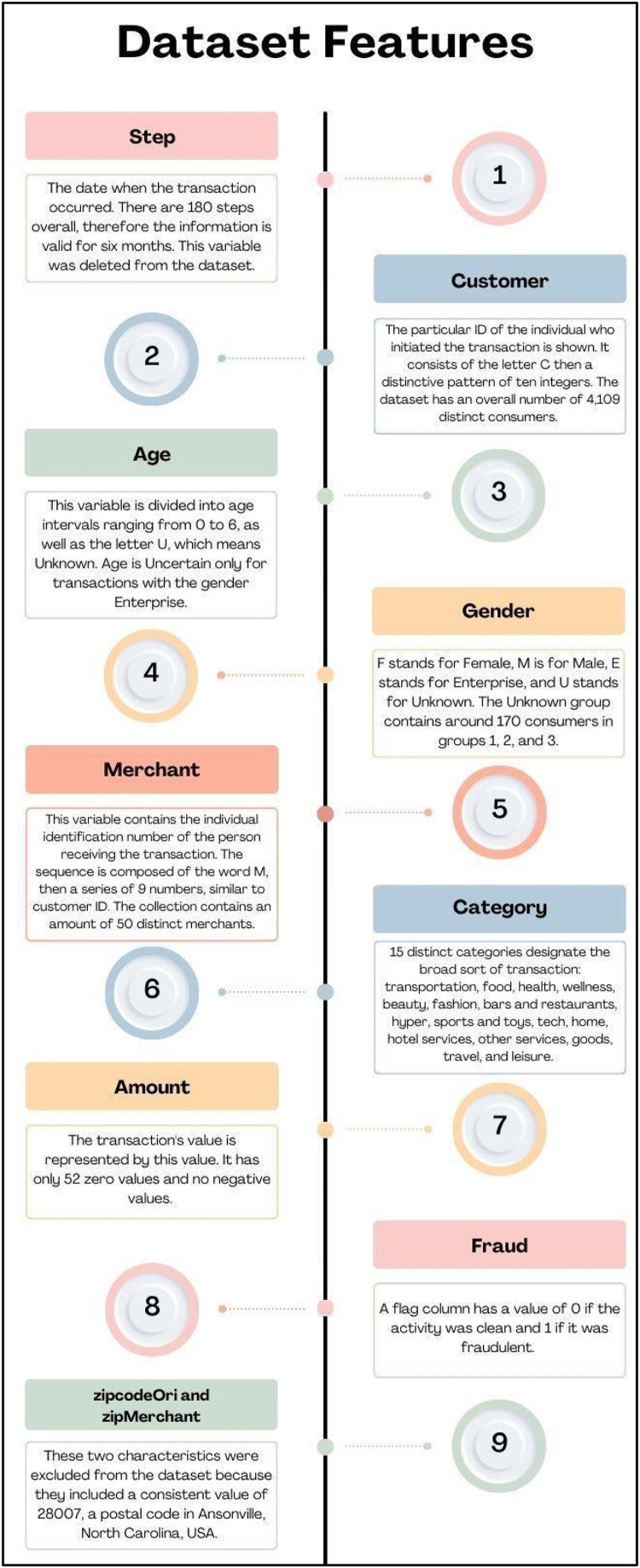
Dataset Features.

Graph databases allow us to extract complex graph-based features that are essential for understanding complex relationships in the data. Specialised graph helper functions are built and integrated to enhance the richness of the dataset. In Fig. 2, on the left side pane, we can see that there are nodes such as Customer, Bank, Merchant, etc. Also the types of relationships among them are shown below such as PAYS, PERFORMS, etc. Now when we query the appropriate graphs of nodes and their relationships, we can get the graphs as shown in Fig. 3 with which we can identify the anomalous transactions. For example, we can identify if there are multiple edges coming into a particular merchant node from a customer node for one single transaction, the transaction could be anomalous. Fig. 3 shows graphical representations from the Neo4J database that depict the PERFORMS relationship, Fig. 4 shows WITH and Fig. 5 shows PAYS relationship between the independent parameters from our data.

Once the graph-based features have been extracted, the next step is to preprocess the data. This is a critical step in preparing the data for machine learning models to be trained. Various techniques are used here to improve the dataset. First, we use one hot encoding for categorical parameters in the dataset [1,2]. This is important because many machine learning algorithms depend on numerical inputs, so one hot encoding converts categorical data into a numerical format while preserving its essence. Second, we use normalisation procedures to make sure that different features have a consistent scale so that no single feature dominates the analysis because of its size. After that, while exploring the data we also came across the unbalanced dataset scenario, where the proportions of data samples of both classes present in the dataset are not comparable, hence if we pass this data to the model as it is, it may end up missing some important features in the dataset, due to data imbalance. Hence, we use SMOTE resampling techniques to balance the data samples for both classes [3].

Now that the dataset is ready for preprocessing, the next step is to select and evaluate the models. Various machine learning models are considered for the analysis, including Support vector machines (SVM), Random forest, k-nearest neighbours (kNN), adaBoost, catboost, etc. [4,5]. Each model has its own unique strengths and uses, so it's important to evaluate its performance consistently. The evaluation metrics for each model are calculated which include accuracy/precision, recall/F1-score/area under the curve (ROC curve), etc.

## Method validation

### Datasets

In this method, we used a dataset called “BankSim”, which simulates bank transactions using an agent-based and a sample of anonymized transactional data from a Spanish bank. The primary use case for BankSim is to generate synthetic data for fraud detection [6]. The generators of this dataset claim that the data generated by BankSim is a useful approximation of the pertinent features of the actual data. The creation of synthetic data for use in fraud detection research is BankSim's primary goal.

Features of the dataset are as below:

As we can see in Fig. 5, the creators of this dataset repeatedly ran BankSim for 180 steps, or around six months, and adjusted the settings to produce a distribution that was nearly accurate enough for testing. In total, they generated 594,643 records, which is 7200 fraudulent transactions and 587,443 legitimate payments were made. The values are different from the real data because this is a randomised simulation [7]. We came to know that in our data there are no null values or duplicated values as well.

One of the challenges we faced in this dataset is its non-balanced nature. Since there are relatively few fraudulent transactions, the training data needs to be carefully picked so that the model can efficiently recognize the patterns in transactions.

### Model parameters and performance metrics

• **Accuracy**

Accuracy is defined as the ratio of the correct classifications to that of the total classifications [[6], [8]]. Accuracy is considered as the gold standard metric for the evaluation of classification algorithms. The formula for accuracy is mentioned in Eq. (1).

(1) $$\mathrm{Accuracy}=\;\frac{\mathrm{TP}+\mathrm{TN}}{\mathrm{TP}+\mathrm{TN}+\mathrm{FP}+\mathrm{FN}}$$

Where TP is True Positive,TN is True Negative, FP is False Positive, and FN is False Negative evaluated from confusion matrix.

• **Precision**

Precision is defined as the ratio of true positives to that of the total positives. Precision is one of the metrics used to analyse the performance of a model in conditions of class imbalance. The formula for precision is mentioned in Eq. (2).

(2) $$\mathrm{Precision}=\frac{\mathrm{TP}}{\mathrm{TP}+\mathrm{FP}}$$

• **Recall**

Recall is defined as the ratio of true positives to that of total samples. Recall is another metric that is used to analyse multiclass classification under the condition of class imbalance. Eq. (3) represents the mathematical formula for recall.

(3) $$\mathrm{Recall}=\frac{\mathrm{TP}}{\mathrm{TP}+\mathrm{FN}}$$

• **F1 score**

**Fig. 6:**
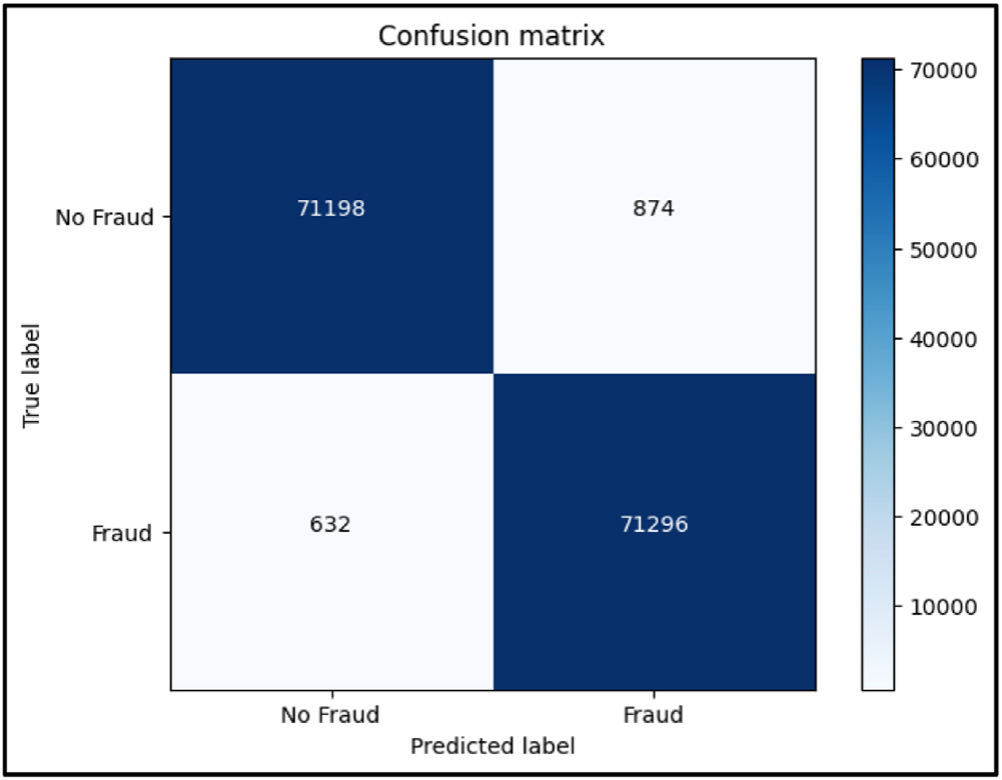
Confusion Matrix for Random forest.

**Fig. 7:**
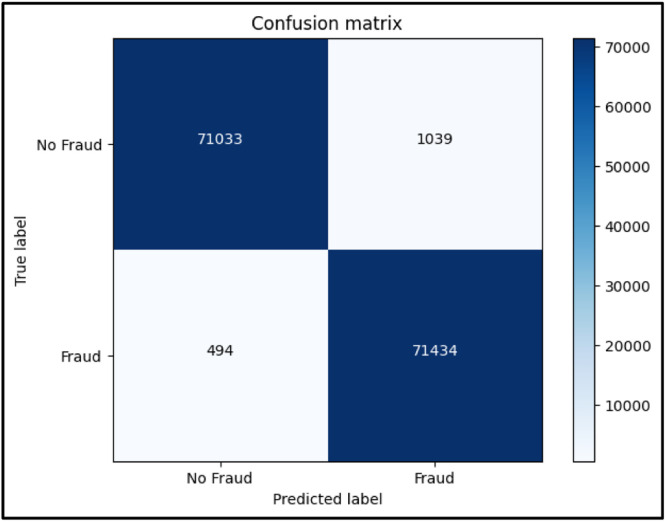
Confusion Matrix for KNN.

**Fig. 8:**
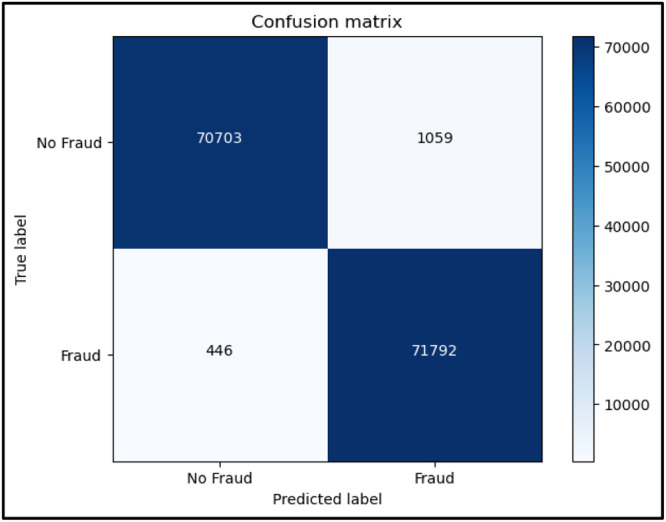
Confusion Matrix for XGBoost.

A popular metric in machine learning for assessing a binary classification model's performance is the F1 score, as described in Eq. (4). When there is an imbalance between the classes, it is especially helpful. The harmonic mean of recall and precision is known as the F1 score. Recall gauges the model's capacity to catch every positive instance, whereas precision assesses the accuracy of positive predictions. By combining these two measures into a single score, the harmonic mean highlights the need to strike a balance between recall and precision. Better overall performance is indicated by a higher score, which goes from 0 to 1. When it's necessary to reduce both false positives and false negatives, the F1 score comes in particularly handy The confusion matrix for Random forest, KNN and XGBoost are shown in Fig. 6, Fig. 7, Fig. 8 respectively.

(4) $$F1\;\mathrm{Score}=2*\;\frac{\mathrm{Precision}*\mathrm{Recall}}{\mathrm{Precision}+\mathrm{Recall}}$$

## Results and discussion

While exploring different ML algorithms, we found that Random Forest, K-Nearest Neighbours, and XGBoost were giving the highest accuracy amongst various algorithms. Some of them are logistic regression, Naive Bayes, linear discriminant analysis, Adaboost, etc. Based on the results we found the highest-performing algorithm was XGBoost giving an accuracy of 99.998%. Moreover as per Table 1 given below, we can see that XGBoost has a minimum time complexity of about 23.9 s as compared to both Random Forest and KNN. Whereas, in the case of KNN, even though it seems to be having the higher accuracy, it also takes up maximum time to run amongst the three of 15 min 14 s. The performance parameters like precision, recall, and F1-score for non-fraud and fraud are shown in Table 2. The accuracy of the models for the prediction of fraud is shown Table 3.

**Table 1:** Time complexity.

	User Time	System Time	Total Time	Wall Time
Random Forest	1 min 13s	132 ms	1 min 13s	1 min 14s
KNN	15 min 13s	1.3 ms	15 min 14s	9 min 44s
XGBoost	23.8s	101 ms	23.9s	15.4s

**Table 2:** Classification report.

	Precision	Recall	F1-Score
0	0.99	0.99	0.99
1	0.99	0.99	0.99

**Table 3:** Methods used and accuracies.

Sr No.	Methodology	Accuracy(%)
1	Random Forest	99.971
2	K Nearest Neighbours	99.983
3	XGBoost	99.998

Following is the confusion matrix:

## Ethics statements

Not applicable.

## Funding statement

This work was supported by the Research Support Fund (RSF) of Symbiosis International (Deemed University), Pune, India.

## CRediT authorship contribution statement

**Ayushi Patil:** Writing – review & editing. **Shreya Mahajan:** Writing – review & editing. **Jinal Menpara:** Writing – review & editing. **Shivali Wagle:** Supervision. **Preksha Pareek:** Supervision. **Ketan Kotecha:** Writing – review & editing.

## Declaration of competing interest

The authors declare that they have no known competing financial interests or personal relationships that could have appeared to influence the work reported in this paper.

## Data Availability

Data will be made available on request.

## References

[1] Can B., Yavuz A.G., Karsligil E.M., Amac Guvensan M. (2020). A closer look into the characteristics of fraudulent card transactions. IEEE Access.

[2] Bin Sulaiman R., Schetinin V., Sant P. (2022). Review of machine learning approach on credit card fraud detection. Hum. Centric Intell. Syst..

[3] Wang C., Zhu H. (2020). Representing fine-grained co-occurrences for behavior-based fraud detection in online payment services. IEEE Trans. Dependable Secur. Comput..

[4] Nanduri J., Liu Y.W., Yang K., Jia Y. (2020). Proceedings of the 2020 Future of Information and Communication Conference (FICC), Advances in Information and Communication.

[5] Khan S., Alourani A., Mishra B., Ali A., Kamal M. (2022). Developing a credit card fraud detection model using machine learning approaches. Int. J. Adv. Comput. Sci. Appl..

[6] Lopez-Rojas E.A., Axelsson S. (2014). Proceedings of the 26th European Modeling and Simulation Symposium, EMSS 2014.

[7] B. Wickramanayake, D. Kapugama Geeganage, C. Ouyang, Y. Xu, “A survey of online card payment fraud detection using data mining-based methods.” arXiv preprint arXiv:2011.14024 (2020).

[8] Kumar S., Kumar H. (2023). Classification of COVID-19 X-ray images using transfer learning with visual geometrical groups and novel sequential convolutional neural networks. MethodsX.

